# Comparative pathogenicity and environmental transmission of recent highly pathogenic avian influenza H5 viruses

**DOI:** 10.1080/22221751.2020.1868274

**Published:** 2021-01-17

**Authors:** Nancy Beerens, Evelien A. Germeraad, Sandra Venema, Eline Verheij, Sylvia B.E. Pritz-Verschuren, Jose L. Gonzales

**Affiliations:** Wageningen University and Research – Wageningen Bioveterinary Research, Lelystad, The Netherlands

**Keywords:** Highly pathogenic avian influenza, H5N8, H5N6, pathogenicity, virus shedding, ducks, Eurasian wigeons, environmental transmission

## Abstract

Strategies to control spread of highly pathogenic avian influenza (HPAI) viruses by wild birds appear limited, hence timely characterization of novel viruses is important to mitigate the risk for the poultry sector and human health. In this study we characterize three recent H5-clade 2.3.4.4 viruses, the H5N8-2014 group A virus and the H5N8-2016 and H5N6-2017 group B viruses. The pathogenicity of the three viruses for chickens, Pekin ducks and Eurasian wigeons was compared. The three viruses were highly pathogenic for chickens, but the two H5N8 viruses caused no to mild clinical symptoms in both duck species. The highest pathogenicity for duck species was observed for the most recent H5N6-2017 virus. For both duck species, virus shedding from the cloaca was higher after infection with group B viruses compared to the H5N8-2014 group A virus. Higher cloacal virus shedding of wild ducks may increase transmission between wild birds and poultry. Environmental transmission of H5N8-2016 virus to chickens was studied, which showed that chickens are efficiently infected by (fecal) contaminated water. These results suggest that pathogenicity of HPAI H5 viruses and virus shedding for ducks is evolving, which may have implications for the risk of introduction of these viruses into the poultry sector.

## Introduction

Highly pathogenic avian influenza (HPAI) viruses form a continuous threat to the poultry industry and to public health. The Eurasian H5N1 lineage (A/Goose/Guangdong/1/1996) first emerged in poultry in China in 1996 [1] and was associated with the first human infections and spillover of the virus to wild birds [2]. The Eurasian HPAI H5 viruses have evolved into 10 genetically distinct HA clades [3] and have spread from Asia to Europe, Africa, and North America. Long-distance migratory birds have played a major role in the global spread of the HPAI H5 viruses. Large numbers of wild waterfowl congregate at breeding sites and disseminate the viruses along their major flyways to wintering sites in Europe [4,5]. In recent years, viruses belonging to the H5 clade 2.3.4.4 have rapidly emerged and evolved into four distinct genetic groups (A–D) [6]. The H5N8 group A viruses were introduced in Europe in 2014, and H5N8 and H5N6 group B viruses were introduced in respectively 2016 and 2017. The outbreaks had large impact on animal health, and millions of domestic birds were involved in the depopulation measures to control the outbreaks. In the Netherlands, multiple commercial poultry farms, hobby holdings, and captive bird facilities became infected between 2014 and 2018 [7–9]. Despite the fact that HPAI H5 viruses were highly pathogenic to gallinaceous poultry, these viruses were not uniformly pathogenic to domestic or wild ducks of different species. The epidemic in 2016–2017 was unusual, as infections with H5N8 group B viruses caused massive mortality of wild waterfowl [10–12]. Whereas no mortality was seen for the earlier H5N8 group A viruses [13,14], and limited mortality of wild birds was observed during the later epidemic caused by H5N6 group B viruses [9].

Experimental infection experiments demonstrated that various wild duck species can become infected with HPAI H5 viruses and excrete virus, without showing clinical symptoms [15–19]. Furthermore, surveillance activities showed the presence of HPAI H5-specific antibodies in various species of wild ducks in Asia and Europe, suggesting that those birds survived infection [20–22]. During the H5N8 group A epidemic in the Netherlands in 2014–2015, the virus was isolated from Eurasian wigeons (*Anas penelope*) exclusively, whereas other species of wild birds were also sampled intensively [21]. In the later H5N8 and H5N6 group B epidemics, viruses were detected in Eurasian wigeons sampled alive or found dead [8, 9, 23]. The Eurasian wigeon is a long-distance migratory dabbling duck with a large wintering population in the Netherlands, around 900,000 birds reside in wetland and lake areas. Eurasian wigeons graze for food on land and are often found in farmland around poultry farms. Therefore, Eurasian wigeons may play an important role in both virus spread over long distances and local spread to poultry farms. Direct contact between poultry and wild birds appears limited [24], and virus transmission likely occurs via indirect contact with infected faeces of wild birds. However, there is limited information on the natural route of introduction into poultry by the contaminated environment.

The recurrence of outbreaks caused by HPAI H5 viruses in wild birds and poultry underscores the need for more insight into the pathogenicity of these viruses for waterfowl and gallinaceous birds. In this study, we experimentally infected chickens (*Gallus gallus*), Pekin ducks (*Anas platyrhynchos domesticus*), and Eurasian wigeons (*Anas penelope)* with a H5N8-2014 group A virus, and group B viruses H5N8-2016 and H5N6-2017. We compared pathogenicity, clinical signs and virus shedding of the different host species after infection with these three recent HPAI H5 viruses. In addition, environmental transmission of the H5N8-2016 virus was studied by the exposure of chickens to contaminated water or bedding material. The results of this study will improve risk assessment and enhance our ability to develop control measures to prevent the introduction of HPAI viruses in the poultry sector.

## Materials and methods

### Ethical statement

The animal experiment and procedures were in accordance with the national regulations on animal experimentation and the project license was approved by the Dutch Central Authority for Scientific Procedures on Animals (CCD) (permit number AVD4010020197787, experiment number 2019.D-0008). The animal procedures were performed conform the guidelines from the European Union directive 2010/63/EU.

### Preparation of the virus inoculum

The viruses used in this study were derived from the index cases in the poultry sector. The whole genome sequences were generated previously and were deposited to the GISAID Database (https://www.gisaid.org): H5N8-2014 (EPI_ISL_168075, A/chicken/Neth/14015531/2014), H5N8-2016 (EPI_ISL_529179, A/duck/Neth/16014829-001005/2016), and H5N6-2017 (EPI_ISL_287906, A/duck/Neth/17017236-001005/2017). Virus stocks were generated by two passages in 9- to 11-day-old specific pathogen free (SPF) embryonated chicken eggs. Full genome sequencing identified a few ambiguous nucleotide positions present in both swabs and egg passages, but no mutations due to passaging. The virus stocks were titrated using standard methods to determine the median egg infectious dose (EID_50_) titers [25]. The virus stocks were diluted in sterile phosphate buffered saline (PBS) immediately prior to use in order to obtain 10^6^ EID_50_/mL inoculum.

### Animals and housing

Six-week-old Specific-Pathogen-Free (SPF) White Leghorn chickens were obtained from MSD Animal Health (Boxmeer, the Netherlands). Six-week-old Pekin ducks were obtained from a commercial breeding farm, and approximately 6-week-old Eurasian wigeons were obtained from several hobby holdings. Eurasian wigeons are seasonal breeders, and therefore the age of the birds likely varied between 4 and 8 weeks old. All animals used in this study were from both sexes. Pekin ducks and Eurasian wigeons tested negative for antibodies against AI virus in serum using ELISA at the day of arrival, and tested negative for AI virus by RT–PCR in swabs on the day of arrival and after an acclimatization period of 7 days. The experiment was performed in biosafety level 3 (BSL 3) facilities at Wageningen Bioveterinary Research (WBVR, Lelystad, the Netherlands). Peking ducks and Eurasian wigeons infected with the same virus were housed in the same room, separated in two floor pens with solid livestock dividing panels. The chickens participating in the infection experiment were housed in one room separated in three floor pens with solid dividing panels. All pens housed 10 birds participating in this study and 12 birds participating in another study until day 4 after infection. The chickens participating in the environmental transmission experiment were individually housed in cages placed in the experimental room. All rooms were temperature-controlled (18.8–22.6°C) and birds were housed under optimal light conditions and humidity, and feed and water were provided ad libitum. Sawdust and some straw were provided as bedding material in the pens. Individual birds were numbered randomly and allocated to an experimental group. Animal caretakers and laboratory personnel were aware of the group allocation during the experiment (not blinded). Eurasian wigeons and Pekin ducks were treated preventively for coccidiosis by the addition of Baycox (Bayer) in the drinking water for 48 h starting at the day of arrival. After the Baycox treatment, swimming ponds were provided to both Pekin ducks and Eurasian wigeons containing approximately 100 litres of tab water. Chickens were provided with perches and pieces of jute as cage enrichment. In the environmental transmission experiment, feed was spread over the bedding material to stimulate contact with the contaminated bedding. The ARRIVE guidelines for reporting animal research were followed [26].

### Experimental design

For the infection experiment, 10 birds of each species were inoculated with either H5N8-2014, H5N8-2016 or H5N6-2017 virus (9 groups). Birds were allowed a 7-day acclimatization period before infection with 0.1 mL of 10^6^ EID_50_/ml inoculum via both intranasal and intratracheal administration (inoculation dose of 10^5.3^ EID_50_ per bird). All birds were observed for 10 days for signs of disease and mortality, and a pathogenicity score was calculated using the scoring protocol of the intravenous pathogenicity index (IVPI) [27]. When a bird reached a humane endpoint, it was euthanized and counted as death on the next day for the calculation of the pathogenicity score. Every day oropharyngeal (OP) and cloacal (CL) swabs were collected from all birds to determine virus shedding, swabs were also collected from dead or euthanized birds (one CL swab for chickens inoculated with H5N8-2016 virus is missing at day 3). Blood was collected at the last day (day 10) to determine antibody production. Bedding and swimming water of all groups of Pekin ducks and Eurasian wigeons were not replaced until day 4 after inoculation. At day 4, swimming water and bedding was collected from the pen housing the Eurasian wigeons infected with the H5N8-2016 virus. Afterwards, swimming water and bedding was removed from all pens and replaced by clean water and bedding, which were then replaced daily until the end of the experiment.

For the environmental transmission study, 24 independent cages were placed in an experimental room. Twenty chickens were individually housed in cages. Ten chickens received contaminated bedding material derived from the pen with the H5N8-2016 infected Eurasian wigeons, and 10 chickens received contaminated swimming water as drinking water. These chickens did not receive other sources of water. Bedding and water were not replaced during the experiment. Two cages contained two sentinel chickens as controls, and two cages contained contaminated bedding material and water without chickens to measure virus survival over time. The chickens were monitored for clinical symptoms and mortality for 7 days after exposure. Everyday OP and CL swabs were taken to determine virus shedding, and blood was collected at the last day of the experiment (day 7) to determine antibody production.

### Antibody detection

Serum was collected from the blood samples and stored at −20°C until testing. For influenza virus-specific antibody detection, serum was tested by anti-NP ELISA as described previously [28]. Hemagglutination inhibition (HI) testing for HA subtype-specific antibodies was performed using standard H5 antigens [27].

#### Virus detection in swabs

Swabs were placed in 2 ml of Tryptose Phosphate Broth (TPB) 2.95% and stored at −70°C until testing. For virus detection, total RNA was extracted from the swabs using the MagNA Pure 96 system (Roche, Basel, Switzerland). Influenza A virus was detected by a quantitative real-time RT–PCR targeting the matrix gene (M-PCR), as described previously [7]. For each virus, a standard curve for virus quantification was taken along that consisted of a 10-fold serial dilution of virus stock with known EID_50_ titer. A standard curve was used to convert the Ct values into equivalent EID_50_ titers and determine PCR efficiency. Amounts of virus detected in swabs are reported as mean equivalent log_10_ EID_50_/mL titers, with a lower detection limit of 10^1.7^ EID_50_/ml.

### Virus detection in bedding and water

The bedding material and the swimming water in the pen housing the H5N8-infected Eurasian wigeons were sampled before collection at day 4 of the infection experiment. Thereafter, the bedding and the water in the two control cages were sampled every day during the environmental transmission experiment (7 days). The bedding was sampled using boot swabs, which were gently placed in containers after 1 min of walking, and were stored at −70°C until analysis. For analysis, 100 ml TPB containing 1% gentamycin was added to the boot swabs, the containers were shaken vigorously after which the suspension was collected. The suspension was filtered, after the final filtering step (0.2 µM filter, Nalgene) a sample of 5 ml was incubated with 1% of penicillin and gentamycin and incubated at room temperature for 1 h. RNA was isolated using MagNA Pure as described above. For sampling of the water, a 1- l sample was collected and filtered directly as described previously [29]. Briefly, the water was filtered using a hydrophilic mixed cellulose esters membrane (MF-Millipore™ Membrane Filter, Merck). Subsequently, RNA was isolated from the filters using the PowerWater® RNA Isolation kit (Qiagen). Viral RNA isolated from the boot swabs and filters was quantified using real-time M-PCR. In-vitro transcribed RNA representing the matrix gene was generated from a plasmid containing a T7-promoter using the MEGAscript™ T7 Transcription Kit (ThermoFisher). A series of 10-fold dilutions of RNA was run simultaneously with the samples to calculate the number of RNA copies per ml. The RNA copies were not converted to equivalent EID_50_ titers, as the amount of viable virus is expected to decrease in time. During the experiment, also 15 ml water samples were taken, which were stored at −70°C. The pH of the water was measured using pH indicator strips (pH 4.5–10, Whatman). The bedding and water samples were analysed for viable virus by inoculation of 200 μl into four SPF embryonated eggs.

### Statistical analysis

#### Infection experiments

One indicator of pathogenicity of the viruses for each of the bird species studied is the level of and time to mortality of an infected host following infection. Comparisons of this induced mortality in time post infection (“survival time”) were done between the different virus infected groups within the same bird species (e.g. among chicken groups) using the log-rank test. Virus shedding was characterized by calculating the area under the curve (AUC) as a measure of total virus shed via OP or CL by each bird during the course of the infection and by calculating the length (duration in days) of shedding via each of these routes (OP or CL). Comparisons of shedding characteristics were done among the different virus inoculated groups within each bird species (e.g. among chicken groups). Mean levels of shedding (AUC) were compared using an ANOVA test, after assessing for normality (Shapiro test) and similarity of variances (Fligner test). If deviations of normality or similarity of variances were observed, comparisons were done using the Kruskal–Wallis test. Length of shedding was compared by fitting parametric survival regression models with a Weibull distribution (distribution that fitted the data best). Analysis was done using the software package R (version 3.6.1) [30]. The library “survival” was used for the survival analysis. The threshold for significance was set to *p* < 0.05.

#### Environmental transmission experiment

For the analysis of the environmental transmission experiment, the environment reproduction number *R*_env_ (the average number of secondary infections caused by a contaminated environment) was calculated using the final size method, where each trial consisted of an environment-susceptible bird and was treated as a one-to-one transmission trial. Maximum likelihood estimation was used to estimate *R*_env_ following the model described by [31] and using the software package R (version 3.6.1) [30].

## Results

### Pathogenicity of HPAI H5 viruses

**Figure 1.**
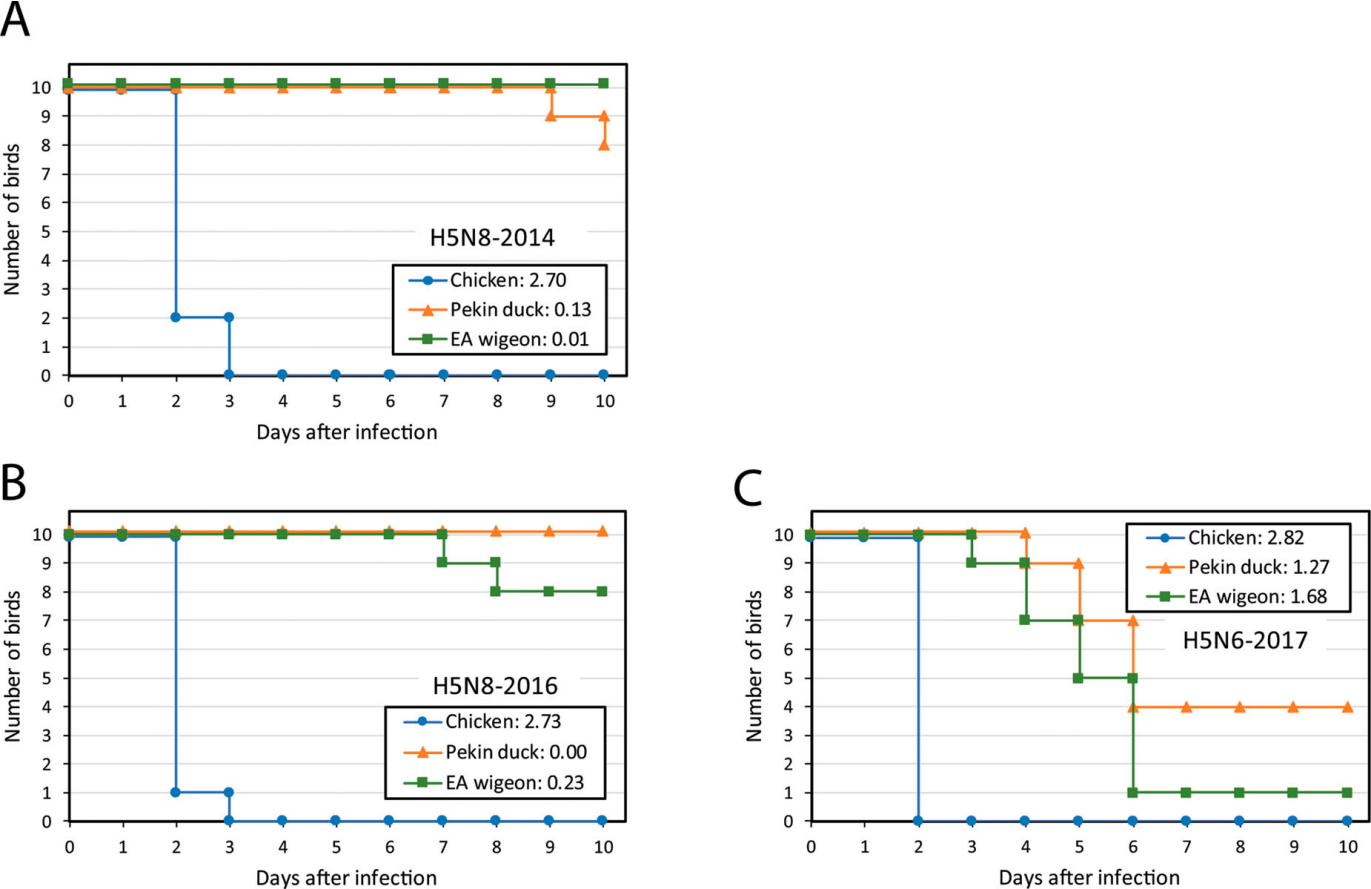
Survival of 6-week-old SPF chickens (blue), Pekin ducks (orange) and Eurasian (EA) wigeons (green) after intratracheal/intranasal infection (doses 10^5.3^ EID_50_/bird) with (A) H5N8-2014, (B) H5N8-2016, and (C) H5N6-2017 viruses. Per group 10 birds were monitored for 10 days for clinical symptoms and mortality. The pathogenicity score was calculated based on OIE criteria for IVPI, and ranges from 0.0 (no pathogenicity) to 3.0 (highest pathogenicity). The pathogenicity scores for the viruses are listed in the legend.

The pathogenicity of recent HPAI H5 viruses for different avian host species was studied by experimental infections of chickens, Pekin ducks and Eurasian wigeons. The viruses were isolated from three index cases in poultry in the Netherlands, H5N8-2014 (H5 clade 2.3.4.4 group A virus), H5N8-2016 and H5N6-2017 (H5 clade 2.3.4.4 group B viruses). Ten birds of each species were infected by intranasal/tracheal inoculation of the viruses and were monitored for 10 days for symptoms of disease and mortality. All surviving birds tested positive for antibodies against AI virus at the end of the experiment (day 10), demonstrating that these birds became infected and seroconverted, except for one Eurasian wigeon inoculated with H5N8-2016 virus. This bird was found positive by PCR analysis, as discussed below. Figure 1 shows the survival curves and the pathogenicity scores for the different bird species after infection with the HPAI H5 viruses. All chickens infected with the HPAI viruses died within 2 or 3 days after infection, resulting in median survival times of 2 days and pathogenicity scores ranging from 2.70 to 2.82. Because all infected chickens died within this short time interval no statistical comparisons of survival were done. Before death, chickens showed only some listlessness and ruffled feathers.

For Pekin ducks, infection with the H5N8-2014 virus resulted in two deaths, and the estimated pathogenicity score was 0.13. Both ducks were listless at day 8, and one duck developed neurological symptoms at day 9 after which it was euthanized. Infection with the H5N8-2016 virus did not result in clinical signs or mortality in Pekin ducks, hence the pathogenicity score was 0.00. Higher mortality was observed in ducks infected with the H5N6-2017 virus, 6 out of the 10 challenged Pekin ducks died between 4 and 6 days after infection, the estimated pathogenicity score was 1.27. The survival time of Pekin ducks infected with the H5N6-2017 virus was significantly shorter than that of ducks infected with the H5N8 viruses (Figure 1). Clinical symptoms started at day 3 after infection and included listlessness, lethargy, ruffled feathers, respiratory symptoms (coughing, sneezing), loss of appetite, nasal and ocular discharge, watery eyes, conjunctivitis and neurological signs.

For the Eurasian wigeons, no mortality was observed after infection with the H5N8-2014 virus, and pathogenicity was limited resulting in a score of 0.01. Diarrhea was observed for one bird at day 9. Infection with the H5N8-2016 virus resulted in two deaths on days 7 and 8 after infection, with no obvious clinical symptoms preceding death. The pathogenicity score was 0.23. The highest pathogenicity score 1.68 was estimated for the H5N6-2017 virus. With this virus nine deaths were observed between days 3 and 6 after infection. The survival of Eurasian wigeons infected with the H5N6-2016 virus was significantly shorter compared to the two H5N8 viruses (Figure 1). Clinical symptoms started at day 3 after infection and included listlessness, ruffled feathers, loss of appetite, diarrhea, and nasal discharge. At day 7, one of the Eurasian wigeons showed neurological signs, illustrated by compulsive swimming of small circles in the pool and impaired consciousness during animal handling. These combined results show that the highest pathogenicity of all three viruses was observed for chickens, whilst lower pathogenicity was observed for the two duck species. For both Pekin ducks and Eurasian wigeons, the highest pathogenicity was observed for the H5N6-2017 virus.

### Virus shedding of birds infected by HPAI H5 viruses

**Figure 2.**
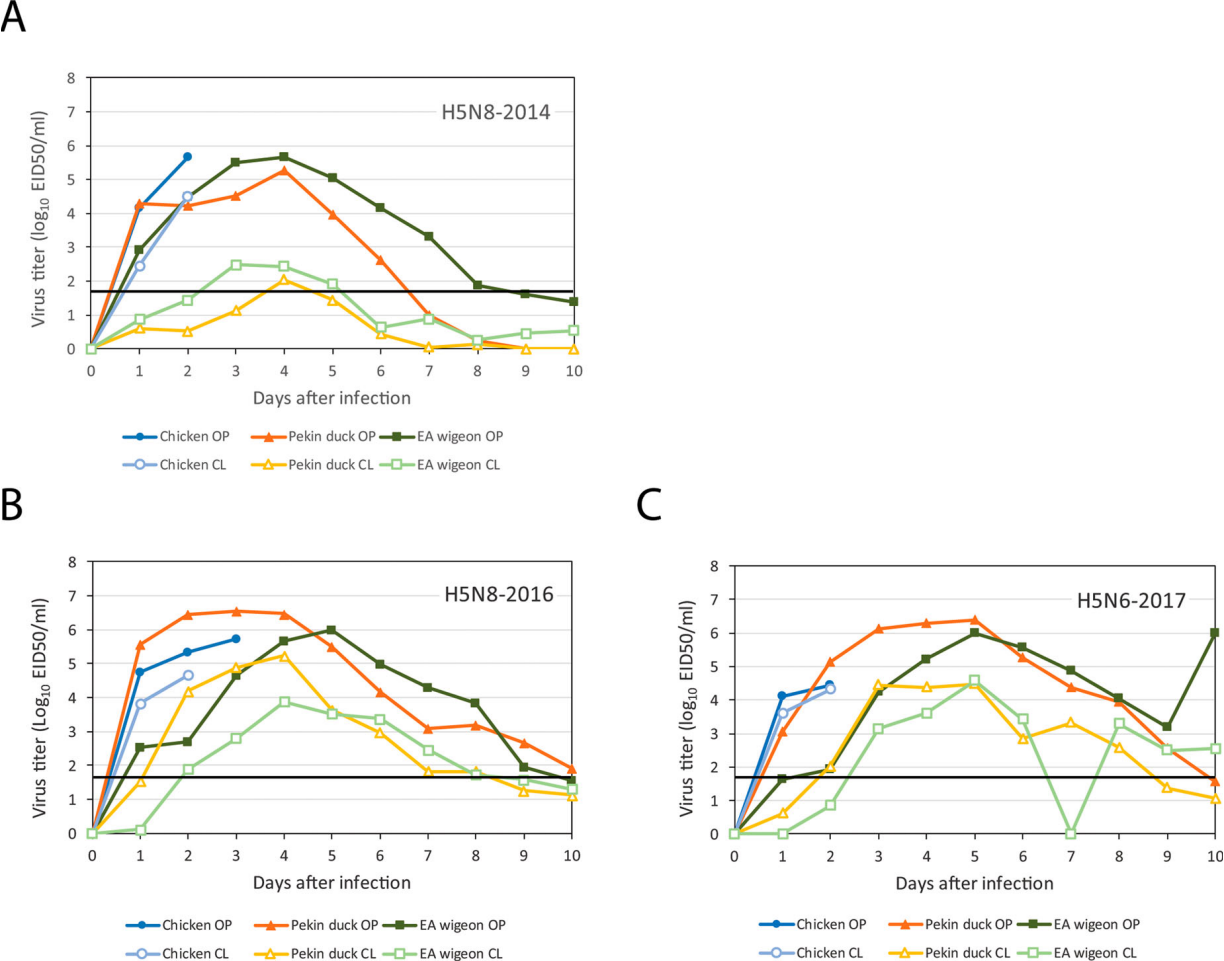
Mean virus shedding (log_10_ EID_50_/ml) after infection with (A) H5N8-2014, (B) H5N8-2016, and (C) H5N6-2017 viruses. Virus shedding was measured in oropharyngeal (OP) and cloacal (CL) swabs collected from chickens (blue), Pekin ducks (orange), and Eurasian (EA) wigeons (orange) during 10 days after infection. The detection limit of 1.7 log_10_ EID_50_/ml is marked by a black line.

To analyse virus shedding of the infected birds, oropharyngeal (OP) and cloacal (CL) swabs were collected every day, and the amount of virus was quantified by real-time RT–PCR. The mean equivalent virus titers measured in the swabs for 10 days after infection are shown in Figure 2, and the distribution of the daily shedding levels of the birds is shown in supplementary Figure S1. Virus shedding was observed for all birds in the study, demonstrating that all birds became infected. For chickens, high levels of OP and CL virus shedding were observed at day 1, until the chickens died at day 2 or 3 after infection for all three HPAI H5 viruses. After infection with the H5N8-2014, high levels of OP virus shedding were observed for both Pekin ducks and Eurasian wigeons, with a peak of virus shedding around day 5 after infection (Figure 2A). However, only limited CL virus shedding was observed for both duck species. After infection with the two group B viruses, high levels of both OP and CL shedding were observed for the two duck species (Figure 2B and C). The onset and peak of virus shedding appear delayed for Eurasian wigeons compared to Pekin ducks. To compare the total amount of virus shed by the birds after HPAI infection, the area under the curve (AUC, log_10_ EID_50_/ml) was calculated (Supplementary Figure S2, Table 1). Furthermore, the duration of virus shedding of the birds was estimated (Table 1). For chickens, the total amount of OP virus shedding was significantly lower for the H5N6-2017 virus (mean AUC 4.0) compared to the two H5N8 viruses (mean AUC 5.3 and 5.4), whilst virus shedding for the H5N6-2017 virus (mean AUC 3.7) was lower than for the H5N8-2016 virus (mean AUC 4.4) only. These differences are likely related with the shorter duration of shedding and survival times observed for the chickens infected with H5N6-2017 virus (see Figure 1). For Pekin ducks, the total amount of OP virus shedding for the two group B viruses (mean AUC 7.13 and 6.70) was significantly increased compared to the H5N8-2014 virus (mean AUC 5.75). The estimated mean duration of OP virus shedding was also significantly longer for the H5N8-2016 virus (12.0 days) compared to the H5N8-2014 virus (6.3 days). The total amount of CL virus shedding for the two group B viruses (mean AUC 6.15 and 5.12) was significantly higher compared to the H5N8-2014 virus (mean AUC 2.73) in Pekin ducks. The estimated mean duration of CL virus shedding was significantly longer for both group B viruses (9.4 and 8.2 days) compared to the H5N8-2014 virus (5.0 days). For Eurasian wigeons, the amount of CL virus shedding was significantly increased for the two group B viruses (AUC 4.96 and 4.55) compared to the H5N8-2014 virus (AUC 3.38). The duration of virus shedding of Eurasian wigeons does not significantly differ between the H5N8-2014 and H5N8-2016 virus, and is shorter for the H5N6-2017 virus due to the shorter survival (see Figure 1). No differences in the total amounts of OP virus shedding were observed for the viruses in Eurasian wigeons. These combined results show that for the two duck species, the amount of cloacal virus shedding is increased for the two later group B viruses, compared to the earlier H5N8-2014 group A virus.

**Table 1.** Virus shedding of HPAI infected birds.

Host	virus	OP swabs		CL swabs	
		AUC	Duration	AUC	Duration
		Titer* ± SD	days (LCI-UCI)	Titer ± SD	Days (LCI-UCI)
Chicken	H5N8-2014	5.33 (0.63)^a^	1.7 (0.6–2.8)^a^	4.06 (0.75)^ab^	1.7 (0.7–2.7)^a^
	H5N8-2016	5.38 (0.37)^a^	1.6 (0.6–2.7)^a^	4.41 (1.50)^a^	1.4 (0.6–2.2)^ab^
	H5N6-2017	4.02 (0.38)^b^	1.1 (0.4–1.8)^b^	3.66 (0.44)^b^	1.1 (0.5–1.8)^b^
Pekin Duck	H5N8-2014	5.75 (0.38)^a^	6.3 (2.7–9.7)^a^	2.73 (1.42)^a^	5.0 (1.6–8.9)^a^
	H5N8-2016	7.13 (0.42)^b^	12.0 (5.2–18.5)^b^	6.15 (0.86)^b^	9.4 (3.1–16.6)^b^
	H5N6-2017	6.70 (0.56)^b^	8.4 (3.6–12.9)^ab^	5.12 (0.73)^b^	8.2 (2.7–14.4)^b^
EA wigeon	H5N8-2014	6.24 (0.51)^a^	11.1 (4.1–18.4)^a^	3.38 (0.44)^a^	8.0 (2.7–14.0)^a^
	H5N8-2016	6.55 (0.86)^a^	9.4 (3.5–15.5)^a^	4.96 (0.77)^b^	9.0 (3.0–15.7)^a^
	H5N6-2017	5.93 (0.38)^a^	5.5 (2.0–9.1)^b^	4.55 (0.75)^b^	5.4 (1.8–9.4)^b^

EA, Eurasian; OP, oropharyngeal; CL, cloacal; AUC, area under the curve; SD, standard deviation; LCI, lower confidence interval; UCI, upper confidence interval.

*Titer in log_10_EID_50_/ml.

^a,b^ Statistically significant difference between the groups.

### Environmental transmission of HPAI H5N8-2016 virus

**Figure 3.**
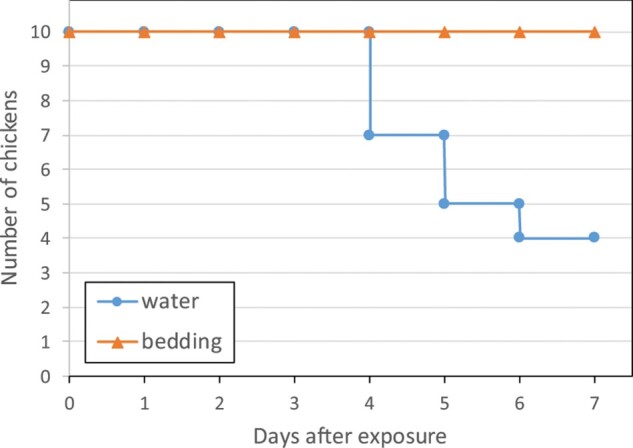
Survival of 6-week-old chickens after exposure to water (blue line) or bedding material (orange line) contaminated by Eurasian wigeons infected with H5N8-2016 virus.

**Figure 4.**
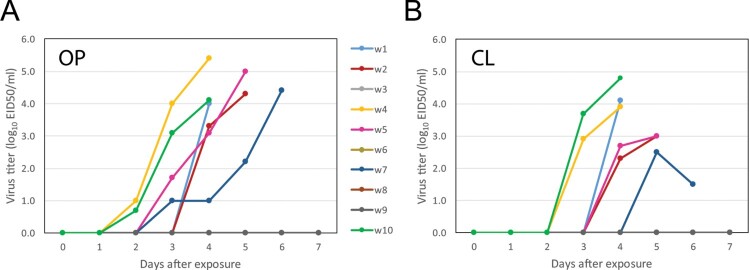
Virus shedding of chickens exposed to contaminated water (w1–w10) for 7 days, measured in (A) oropharyngeal (OP) swabs and (B) cloacal (CL) swabs.

Environmental transmission of the HPAI H5N8-2016 virus was studied to obtain more information on the natural route of virus transmission to poultry by indirect contact with infected wild birds. We therefore collected bedding material and swimming water from the pen housing the infected Eurasian wigeons. The materials were collected at day 4 after infection, at the peak of virus shedding from the cloaca (Figure 2). Ten chickens individually housed in cages received contaminated bedding, and 10 chickens received contaminated water for drinking. The experiment included two cages with a pair of sentinel chickens, and two cages with contaminated water and bedding only (no chickens). The survival curves for the chickens for 7 days after exposure to contaminated bedding or water are shown in Figure 3. All 10 chickens survived after exposure to bedding, whilst 6 chickens died after exposure to water. To analyse virus infection, OP and CL swabs were collected from the chickens every day, and blood was collected at the end of the experiment (day 7). Figure 4 shows that virus shedding was observed for the six chickens that died during the experiment after exposure to contaminated water. For two chickens OP virus shedding was already observed at day 2, for two chickens at day 3, and for two at day 4 after exposure. Virus shedding from the cloaca initiated approximately 1 day after oropharyngeal shedding started. No virus shedding was observed neither for the four chickens still alive at the end of the experiment, nor for chickens exposed to bedding or for sentinel chickens. All surviving chickens tested negative for AI antibodies at the end of the experiment. Based on the number of transmission events observed (6 out 10), and the assumption that virus decay in the environment during the 7 days of exposure was very low in water (as described below), the estimated *R*_env_ for environmental transmission of HPAI via water was 3.0 (95% confidence interval (CI): 1.8–4.2) and via bedding was 0.0 (95% CI: 0.0–0.77). These results show that environmental transmission of HPAI virus to chickens via contaminated water is efficient (*R*_env_ > 1), whereas transmission via contaminated bedding material is less likely to happen (*R*_env_ < 1).

### Persistence of HPAI H5N8-2016 virus in water and bedding material

**Figure 5.**
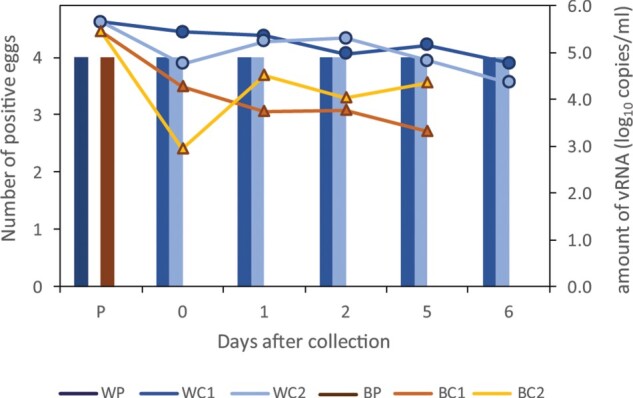
Persistence of virus in water and bedding material in the two control cages. The viability of the virus was tested by inoculation into four embryonated eggs (columns), and the amount of viral RNA was determined by real-time RT-PCR (line graph). Samples of water and bedding material were tested pre-collection (P) in the pen housing the Eurasian wigeons, and at days 0, 1, 2, and 5 of the environmental transmission experiment. Water samples were also tested on day 6. WP: water pre-collection, WC1: water control cage 1, WC2: water control cage 2, BP: bedding pre-collection BC1: bedding control cage 1, BC2: bedding control cage 2.

The persistence of the virus in water and bedding material was studied. Samples were taken pre-collection of materials from the pen housing the infected Eurasian wigeons (day 4), and subsequently from the two control cages during the 7 days of the environmental transmission experiment. The pH of the water samples was consistently 7.5 during the experiment, and the average temperature in the experimental room was 21.1°C. Bedding and water samples were analysed by real-time RT–PCR for the presence of AI virus, as is shown in Figure 5 (line graph). Viral RNA could be detected in bedding samples (10^5.5^ vRNA copies/ml) and water samples (10^5.7^ vRNA copies/ml) collected from the pen housing the Eurasian wigeons. The amount of viral RNA detected in the two control cages declined during the experiment, approximately 1000-fold for the bedding samples at day 5, and 100-fold for the water samples at day 6. To test whether the virus detected by PCR is still infectious, samples of the water and bedding material were inoculated into four embryonated eggs (Figure 5, columns). All four eggs tested positive for virus isolation after inoculation with bedding or water samples collected from the pen housing the infected Eurasian wigeons, demonstrating that the environmental transmission experiment was started with infectious virus. Virus was isolated from all eggs inoculated with water samples that were collected from the two control cages during the experiment. The virus thus remained infectious in water until day 6, which was the last day tested. However, samples taken from bedding material during the experiment did not contain viable virus. These results demonstrate a long persistence of infectious virus in water compared to bedding material.

## Discussion

In this study, we characterize three representative HPAI H5 viruses isolated from the index cases in poultry in the Netherlands. The H5H8-2014 virus belongs to H5 clade 2.3.4.4 group A, whereas the H5N8-2016 and H5N6-2017 viruses are classified as group B viruses. The pathogenicity of the three virus strains was directly compared in three avian host species: chickens and Pekin ducks (the most common domestic galliform and anseriform species), and Eurasian wigeons (as a model species for wild birds). Consistent with the pathogenicity of other clade 2.3.4.4 H5 viruses, the three viruses caused 100% mortality in experimentally infected chickens, with a median survival time of 2 days. For Pekin ducks, however, differences were observed between the three viruses. Pekin ducks infected with the H5N8-2014 and 2016 viruses showed very limited to no clinical symptoms. Mortality was 20% for the H5N8-2014 virus, and 0% for H5N8-2016 virus, resulting in low pathogenicity scores (0.13 and 0.00 respectively). After infection of Pekin ducks with the H5N6-2017 virus, symptoms of disease were observed starting 3 days after infection, and 60% mortality was observed resulting in a pathogenicity score of 1.27. The survival of Pekin ducks after infection with H5N6-2017 virus was significantly shorter compared to the two earlier H5N8 viruses. Previously performed IVPI studies in 6-week-old Pekin ducks demonstrated lower pathogenicity for the H5N8-2014 virus (IVPI score 1.87) compared to H5N8-2016 and H5N6-2017 viruses (IVPI scores 2.99 and 3.00 respectively) [9]. Direct injection of viruses into the blood stream thus resulted in a higher pathogenicity scores for the viruses, in particular for the H5N8-2016 virus, compared to intranasal/intratracheal administration. Previous studies on recent HPAI H5 clade 2.3.4.4 viruses revealed a range of pathogenicity scores for wild and domestic ducks, with mortality varying between 0% and 20% [17, 18, 32–36]. Variation may be caused by differences in virus stains, inoculation route, or bird species and age. This underlines the importance of the direct comparison of virus strains in different bird species, as was performed in this study. We studied pathogenicity in 6-week-old birds, which is the age of chickens standardly used in IVPI experiments. Mortality and virus shedding may be reduced in older birds, in particular when they experienced previous infections with avian influenza viruses. The limited clinical signs observed upon the infection of Pekin ducks with the different HPAI H5 viruses may have prevented early detection of the outbreaks on commercial duck farms. Consistent with this, a retrospective study showed that during the 2014–2018 outbreaks, clinical signs were seldomly observed on farms before mortality increases [37].

For Eurasian wigeons, infection with the two H5N8 viruses resulted in very limited clinical symptoms. Mortality was 0% for the H5N8-2014 virus, and 20% for H5N8-2016 virus, resulting in low pathogenicity scores (0.01 and 0.23 respectively). The mortality caused by the H5N6-2017 virus was 90%, and the survival time was significantly shorter than that observed for the two H5N8 viruses. Symptoms of disease started 3 days after infection, resulting in a pathogenicity score of 1.68. This demonstrates that the most recent HPAI H5 virus, the H5N6-2017 virus, is most pathogenic for both the domestic Pekin duck and the wild Eurasian wigeon. High levels of virus shedding were measured for the two duck species after infection with the HPAI H5 viruses, even when no clinical symptoms were observed. Infection with H5N8-2014 virus mainly resulted in shedding from the oropharynx, whilst after infection with the two group B viruses virus shedding from both oropharynx and cloaca was observed. The total amount of virus shed from the cloaca of Pekin ducks and Eurasian wigeons was significantly increased for the two group B viruses compared to the H5N8-2014 virus (Table 1). Virus transmission between wild birds is through to occur via the fecal–oral route, whereby infected birds shed viruses in feces, and others are infected by feeding in virus-contaminated water. The low cloacal shedding of duck species after infection with the H5N8-2014 virus may have limited transmission of the virus among wild birds, resulting in a low prevalence of the virus in the wild bird population. This is consistent with the fact that no wild bird mortality was observed during the epidemic in 2014–2015, and the virus was only detected in a few living wild birds. The increased cloacal shedding observed for the later H5N8-2016 virus likely resulted in efficient transmission of this virus in the wild bird population. The massive mortality of Eurasian wigeons and other wild waterfowl observed during the 2016–2017 epidemic may be caused by the slightly increased pathogenicity of the H5N8-2016 virus (compared to the H5N8-2014 virus), combined with a high prevalence of the virus in the wild bird population due to increased cloacal shedding [10, 23]. Increased cloacal virus shedding was also observed for the two duck species after infection with the H5N6-2017 virus. However, the H5N6-2017 virus was also more pathogenic for duck species than the two H5N8 viruses, causing significantly increased mortality. This may have hampered long-distance travelling of infected wild birds, thereby limiting the spread of the virus along the migration routes. The limited number of infected wild birds found dead during the H5N6 epidemic in 2017–2018 in the Netherlands suggests the prevalence of the virus was low amongst migratory wild birds. Furthermore, wild bird surviving the previous H5N8 virus infections may have acquired immunity against re-infection with related HPAI H5 viruses. Evidence for the presence of HPAI H5-specific antibodies in various species of wild ducks was obtained during surveillance studies in Asia and Europe [20–22]. Moreover, an experimental infection study with Pekin ducks and mallards showed that ducks surviving infection with a H5N8 group B virus were protected from re-infection with the homologous virus [38]. The pre-existing immunity in the wild bird population thus may have further hampered the spread of genetically related H5N6 group B virus during the 2017–2018 epidemic. In 2020, a novel reassortant HPAI H5N8 group B virus was detected in wild birds found dead in Eastern-Europe and Germany. This virus was genetically related to the H5N8 group B viruses detected in 2016, but obtained novel PB1 and NP segments [39]. Wild bird mortality was limited, and the virus did not spread to other countries in Europe, suggesting a low prevalence in the wild bird population. However, experimental infection studies with this novel virus will be required to evaluate pathogenicity and virus shedding. Active surveillance for HPAI viruses and antibodies in wild birds is essential to provide more information on the prevalence of the viruses in wild bird populations during future epidemics.

This study showed that the pathogenicity of the H5N6-2017 virus is increased for both Pekin ducks and Eurasian wigeons compared to the earlier H5N8 viruses.. Pekin ducks are a domestic duck breed descended from the wild mallard (*Anas platyrhynchos*), a species of migrating dabbling ducks of the *Anatidae* family, that also includes Eurasian wigeons. Whether the pathogenicity of the H5N6-2017 virus is also increased for other members of the Anatidae family remains to be determined, as differences between specific duck species have been reported in a recent study for an HPAI H5N8 group A virus [16]. The mechanism underlying the enhanced pathogenicity of the H5N6-2017 virus for the two duck species is not well understood, and further studies will be required to provide more insight. Likely, besides the HA gene of the viruses, also internal genes contribute to pathogenicity. Phylogenetic analysis of the full genome sequences of the two group B viruses revealed that the H5N6-2017 virus obtained novel PB2 and PA segments, besides the novel NA segment [40]. Genetic analysis identified four amino acid changes in the HA protein, and several in other proteins, but none of them have been previously implicated in pathogenicity (see Supplementary Table S1). Further studies will be required to identify the genetic determinants of increased pathogenicity of the H5N6-2017 virus. The evolution of H5 viruses towards enhanced duck pathogenicity may be driven by prolonged circulation of these viruses in poultry populations in Asia, whereby vaccination may have provided a strong selection pressure. Viruses that evolved in vaccinated poultry populations may have been introduced back into wild birds. Alternatively, the virus may have evolved in wild birds after repeated exposure to HPAI H5 clade 2.3.4.4 viruses. Widespread immunity against HPAI H5 stains in the wild bird population may have driven the evolution of the viruses towards higher pathogenicity.

Poultry can become infected with HPAI viruses by the fecal-oral route, when feeding in an environment contaminated with infected wild bird feces. Therefore, increased cloacal shedding by wild ducks infected with HPAI H5 clade 2.3.4.4 group B viruses likely enhances the risk of introduction of these viruses into the commercial poultry sector. In agreement with this, more outbreaks were reported on commercial poultry farms and hobby holdings during the H5N8 group B virus epidemic in 2016–2017 [8], than during the H5N8 group A epidemic in 2014–2015 in the Netherlands [7]. The number of outbreaks in poultry during the H5N6 group B virus epidemic in 2017–2018 was limited compared to the previous H5N8 group B epidemic, which may have been caused by a decreased prevalence of the virus in the wild bird population [9]. In this study, we investigated environmental transmission of the HPAI H5N8-2016 virus to chickens by water or bedding material contaminated by infected Eurasian wigeons. We show that virus transmission to chickens via contaminated water was efficient, as the estimated *R*_env_ was higher than 1 (lower 95%CI was 1.7). Virus transmission via contaminated bedding material was not observed indicating a low probability of transmission via this route (*R*_env_ < 1). The virus in the bedding material was rapidly inactivated, whereas the virus in water remained infectious for at least 6 days at an average temperature of 21.1°C. The viability of low pathogenic avian influenza (LPAI) viruses in poultry bedding was investigated previously, and dependent on the type of bedding this viability was between 1 and 3 days [41], which is consistent with our results using saw dust as bedding material. Previous studies also showed long-term survival of HPAI H5N1 virus in water, for 14 days at 20°C, up to 60 days at 4°C [42,43]. Few experimental studies have addressed environmental transmission of avian influenza viruses, but these reported similar results. Transmission of LPAI viruses to chickens was reported to occur via water [44,45], and transmission of HPAI H5N1 virus via water and soil [46,47]. The risk of introduction of avian influenza for outdoor-layer farms is six times higher than for indoor-layer farms [48], and the risk in the winter months is four times higher than in the summer months in the Netherlands [49]. In the winter, large numbers of migratory wild birds are present in the Netherlands, but also increased rainfall and lower temperatures will provide optimal circumstances for long-term survival of virus in the environment. The virus titers that accumulated in the swimming water of the infected Eurasian wigeons in this experiment may have exceeded those expected in natural situations with wild birds. However, they may not be unrealistic for small bodies of water or puddles in free-range areas after heavy rainfall. Observational studies have shown that different species of wild ducks visit the free-range area at night for swimming and foraging in puddles of water, whereby direct contact with chickens did not occur [24]. The infection-pressure around poultry farms may build-up due to repeated visits of HPAI infected wild birds, and the long-term survival of the virus in water at lower temperatures. Hence indirect transmission via a contaminated environment may be a main route for infection of poultry, although several poultry farms without free-range area became infected during the HPAI H5 epidemics in the Netherlands in 2014–2018 [7–9], suggesting that other routes of transmission may have been involved. The contribution of environmental transmission, particularly via water, to outbreaks in poultry may be substantial and therefore warrants consideration in models predicting outbreak probability, as previously suggested [50, 51].

## Supplementary Material

FigS2_AUCboxplot_vs2-1_editable.jpg

FigS1_Shedding_Jose_vs2-1-editable.jpg

TableS1_mutations_revised.docx

## References

[1] Xu X, Subbarao K, Cox NJ, et al. Genetic characterization of the pathogenic influenza A/Goose/Guangdong/1/96 (H5N1) virus: similarity of its hemagglutinin gene to those of H5N1 viruses from the 1997 outbreaks in Hong Kong. Virology. 1999;261:15–19. doi:10.1006/viro.1999.9820. 10484749

[2] Ellis TM, Barry B R, Bissett LA, et al. Investigation of outbreaks of highly pathogenic H5N1 avian influenza in waterfowl and wild birds in Hong Kong in late 2002. Avian Pathol J W.V.P.A. 2004;33:492–505. doi:10.1080/03079450400003601 15545029

[3] World Health Organization. Revised and updated nomenclature for highly pathogenic avian influenza A (H5N1) viruses. Influenza Other Respir Viruses. 2014;8:384–388. doi:10.1111/irv.12230 24483237 PMC4181488

[4] Global Consortium for H5N8 and Related Influenza viruses. Role for migratory wild birds in the global spread of avian influenza H5N8. Science. 2016;354:213–217. doi:10.1126/science.aaf8852 27738169 PMC5972003

[5] Ramey AM, Reeves AB, TeSlaa JL, et al. Evidence for common ancestry among viruses isolated from wild birds in Beringia and highly pathogenic intercontinental reassortant H5N1 and H5N2 influenza A viruses. Infect Genet Evol J Molec Epidemiol Evolution Genet Infect Dis. 2016;40:176–185. doi:10.1016/j.meegid.2016.02.035 26944444

[6] Lee DH, Bahl J, Torchetti MK, et al. Highly pathogenic avian influenza viruses and generation of novel reassortants, United States, 2014–2015. Emerg Infect Dis.. 2016;22:1283–1285. doi:10.3201/eid2207.160048 27314845 PMC4918163

[7] Bouwstra RJ, et al. Phylogenetic analysis of highly pathogenic avian influenza A(H5N8) virus outbreak strains provides evidence for four separate introductions and one between-poultry farm transmission in the Netherlands, November 2014. Euro Surveill Eur Commun Dis Bull. 2015;20:31–42. 10.2807/1560-7917.es2015.20.26.21174 26159311

[8] Beerens N, Heutink R, Bergervoet SA, et al. Multiple reassorted viruses as cause of highly pathogenic avian influenza A(H5N8) virus epidemic, the Netherlands, 2016. Emerging Infect Dis.. 2017;23:1974–1981. doi:10.3201/eid2312.171062 PMC5708218 29148396

[9] Beerens N, Heutink R, Pritz-Verschuren S, et al. Genetic relationship between poultry and wild bird viruses during the highly pathogenic avian influenza H5N6 epidemic in the Netherlands, 2017-2018. Transbound Emerg Dis. 2019;66:1370–1378. doi:10.1111/tbed.13169 30874364 PMC6849594

[10] Kleyheeg E, Slaterus R, Bodewes R, et al. Deaths among wild birds during highly pathogenic avian influenza A(H5N8) virus outbreak, the Netherlands. Emerging Infect. Dis.. Dec. 2017;23(12):2050–2054. 10.3201/eid2312.171086 PMC5708256 29148372

[11] Li M, Bi Y, Sun J, et al. Highly pathogenic avian influenza A(H5N8) virus in wild migratory birds, Qinghai Lake, China. Emerg Infect Dis.. 2017;23:637–641. doi:10.3201/eid2304.161866 28169827 PMC5367427

[12] Pohlmann A, Starick E, Harder T, et al. Outbreaks among wild birds and domestic poultry caused by reassorted influenza A(H5N8) clade 2.3.4.4 viruses, Germany, 2016. Emerging Infect Dis.. 2017;23:633–636. doi:10.3201/eid2304.161949 PMC5367393 28055819

[13] Alarcon P, Brouwer A, Venkatesh D, et al. Comparison of 2016–17 and previous epizootics of highly pathogenic avian influenza H5 Guangdong lineage in Europe. Emerg Infect Dis.. 2018;24:2270–2283. doi:10.3201/eid2412.171860 30457528 PMC6256410

[14] Lee DH, Bertran K, Kwon JH, et al. Evolution, global spread, and pathogenicity of highly pathogenic avian influenza H5Nx clade 2.3.4.4. J Vet Sci. 2017;18:269–280. doi:10.4142/jvs.2017.18.S1.269 28859267 PMC5583414

[15] Keawcharoen J, van Riel D, van Amerongen G, et al. Wild ducks as long-distance vectors of highly pathogenic avian influenza virus (H5N1). Emerg Infect Dis.. 2008;14:600–607. doi:10.3201/eid1404.071016 18394278 PMC2570914

[16] van den Brand JMA, et al. Wild ducks excrete highly pathogenic avian influenza virus H5N8 (2014–2015) without clinical or pathological evidence of disease. Emerg Microbes Infect. 2018;7:67. doi:10.1038/s41426-018-0070-9 29670093 PMC5906613

[17] Kwon JH, Noh YK, Lee DH, et al. Experimental infection with highly pathogenic H5N8 avian influenza viruses in the Mandarin duck ( Aix galericulata ) and domestic pigeon ( Columba livia domestica ). Vet Microbiol. 2017;203:95–102. doi:10.1016/j.vetmic.2017.03.003 28619174

[18] Son K, Kim YK, Oem JK, et al. Experimental infection of highly pathogenic avian influenza viruses, clade 2.3.4.4 H5N6 and H5N8, in Mandarin ducks from South Korea. Transbound Emerg Dis. 2018;65:899–903. doi:10.1111/tbed.12790 29266850

[19] Kang HM, Lee EK, Song BM, et al. Experimental infection of mandarin duck with highly pathogenic avian influenza A (H5N8 and H5N1) viruses. Vet Microbiol. 2017;198:59–63. doi:10.1016/j.vetmic.2016.12.005 28062008

[20] Jeong J, Kang HM, Lee EK, et al. Highly pathogenic avian influenza virus (H5N8) in domestic poultry and its relationship with migratory birds in South Korea during 2014. Vet Microbiol. 2014;173:249–257. doi:10.1016/j.vetmic.2014.08.002 25192767

[21] Poen MJ, et al. Lack of virological and serological evidence for continued circulation of highly pathogenic avian influenza H5N8 virus in wild birds in the Netherlands, 14 November 2014 to 31 January 2016. Eur Commun Disease Bull. 2016;21. doi:10.2807/1560-7917.ES.2016.21.38.30349 PMC5073202 27684783

[22] Gilbert M, Koel BF, Bestebroer TM, et al. Serological evidence for non-lethal exposures of Mongolian wild birds to highly pathogenic avian influenza H5N1 virus. PLoS One. 2014;9:e113569. doi:10.1371/journal.pone.0113569 25502318 PMC4266605

[23] Poen MJ, et al. Local amplification of highly pathogenic avian influenza H5N8 viruses in wild birds in the Netherlands, 2016 to 2017. Eur Commun Dis Bull. 2018;23. doi:10.2807/1560-7917.ES.2018.23.4.17-00449 PMC5801337 29382414

[24] Elbers ARW, Gonzales JL. Quantification of visits of wild fauna to a commercial free-range layer farm in the Netherlands located in an avian influenza hot-spot area assessed by video-camera monitoring. Transbound Emerg Dis. 2020;67:661–677. doi:10.1111/tbed.13382 31587498 PMC7079184

[25] Spackman E, Killian ML. Avian influenza virus isolation, propagation, and titration in embryonated chicken eggs. Methods Mol Biol. 2014;1161:125–140. doi:10.1007/978-1-4939-0758-8_12 24899426

[26] Percie du Sert N, Hurst V, Ahluwalia A, et al. The ARRIVE guidelines 2.0: Updated guidelines for reporting animal research. PLoS Biol. 2020;18:e3000410. doi:10.1371/journal.pbio.3000410 32663219 PMC7360023

[27] World Organisation for Animal Health. OIE. *Terrestrial manual, Chapter 2.3.4 Avian influenza*. 2015.

[28] Germeraad E, Achterberg R, Venema S, et al. The development of a multiplex serological assay for avian influenza based on luminex technology. Methods. 2019;158:54–60. doi:10.1016/j.ymeth.2019.01.012 30707951

[29] Germeraad EA, Elbers ARW, de Bruijn ND, et al. Detection of low pathogenic avian influenza virus subtype H10N7 in poultry and environmental water samples during a clinical outbreak in commercial free-range layers, Netherlands 2017. Front Vet Sci. 2020;7:237.doi:10.3389/fvets.2020.00237 32478107 PMC7232570

[30] R Core Team. R: A language and environment for statistical computing. Vienna: R Foundation for Statistical Computing; 2019. https://www.r-project.org/

[31] Velthuis AG, de Jong MC, de Bree J, et al. Quantification of transmission in one-to-one experiments. Epidemiol Infect. 2002;128:193–204. doi:10.1017/s0950268801006707 12002537 PMC2869812

[32] Zhao K, Gu M, Zhong L, et al. Characterization of three H5N5 and one H5N8 highly pathogenic avian influenza viruses in China. Vet Microbiol. 2013;163:351–357. doi:10.1016/j.vetmic.2012.12.025 23375651

[33] Lee DH, Kwon JH, Noh JY, et al. Pathogenicity of the Korean H5N8 highly pathogenic avian influenza virus in commercial domestic poultry species. Avian Pathol J WVPA. 2016;45:208–211. doi:10.1080/03079457.2016.1142502 26814367

[34] Sun H, Pu J, Hu J, et al. Characterization of clade 2.3.4.4 highly pathogenic H5 avian influenza viruses in ducks and chickens. Vet Microbiol. 2016;182:116–122. doi:10.1016/j.vetmic.2015.11.001 26711037

[35] Grund C, Hoffmann D, Ulrich R, et al. A novel European H5N8 influenza A virus has increased virulence in ducks but low zoonotic potential. Emerg Microbes Infect. 2018;7:1–14. doi:10.1038/s41426-018-0130-1 30026505 PMC6053424

[36] Uchida Y, Mine J, Takemae N, et al. Comparative pathogenicity of H5N6 subtype highly pathogenic avian influenza viruses in chicken, Pekin duck and Muscovy duck. Transbound Emerg Dis. 2019;66:1227–1251. doi:10.1111/tbed.13141 30720248

[37] Schreuder J, et al. Highly pathogenic avian influenza subtype H5Nx clade 2.3.4.4 outbreaks in Dutch poultry farms, 2014–2018: clinical signs and mortality. Transbound Emerg Dis. 2020. doi:10.1111/tbed.13597 PMC8048556 32418364

[38] Koethe S, Ulrich L, Ulrich R, et al. Modulation of lethal HPAIV H5N8 clade 2.3.4.4B infection in AIV pre-exposed mallards. Emerg Microbes Infect. 2020;9:180–193. doi:10.1080/22221751.2020.1713706 31969057 PMC7006783

[39] King J, et al. Novel HPAIV H5N8 reassortant (Clade 2.3.4.4b) detected in Germany. Viruses. 2020;12. doi:10.3390/v12030281 PMC7150876 32143363

[40] Beerens N, Koch G, Heutink R, et al. Novel highly pathogenic avian influenza A(H5N6) virus in the Netherlands, December 2017. Emerging Infect. Dis.. 2018;24:770–773. doi:10.3201/eid2404.172124 PMC5875252 29381134

[41] Reis A, Stallknecht D, Ritz C, et al. Tenacity of low-pathogenic avian influenza viruses in different types of poultry litter. Poult Sci. 2012;91:1745–1750. doi:10.3382/ps.2011-01625 22802163

[42] Dovas CI, Papanastassopoulou M, Georgiadis MP, et al. Detection and quantification of infectious avian influenza A (H5N1) virus in environmental water by using real-time reverse transcription-PCR. Appl Environ Microbiol. 2010;76:2165–2174. doi:10.1128/AEM.01929-09 20118369 PMC2849232

[43] Domanska-Blicharz K, Minta Z, Smietanka K, et al. H5n1 high pathogenicity avian influenza virus survival in different types of water. Avian Dis. 2010;54:734–737. doi:10.1637/8786-040109-ResNote.1 20521724

[44] VanDalen KK, Franklin AB, Mooers NL, et al. Shedding light on avian influenza H4N6 infection in mallards: modes of transmission and implications for surveillance. PLoS One. 2010;5:e12851. doi:10.1371/journal.pone.0012851 20877466 PMC2942899

[45] Claes G, Marche S, Dewulf J, et al. An experimental model to analyse the risk of introduction of a duck-originated H5 low-pathogenic avian influenza virus in poultry through close contact and contaminative transmission. Epidemiol Infect. 2014;142:1836–1847. doi:10.1017/S0950268813002793 24252718 PMC9151305

[46] Gutierrez RA, Buchy P. Contaminated soil and transmission of influenza virus (H5N1). Emerg Infect. Dis.. 2012;18:1530–1531.doi:10.3201/eid1809.120402 22931910 PMC3437732

[47] Forrest HL, Kim JK, Webster RG. Virus shedding and potential for interspecies waterborne transmission of highly pathogenic H5N1 influenza virus in sparrows and chickens. J Virol. 2010;84:3718–3720. doi:10.1128/JVI.02017-09 20089659 PMC2838142

[48] Bouwstra R, Gonzales JL, de Wit S, et al. Risk for low pathogenicity avian influenza virus on poultry farms, the Netherlands, 2007–2013. Emerg Infect Dis.. 2017;23:1510–1516. doi:10.3201/eid2309.170276 28820139 PMC5572893

[49] Gonzales JL, et al. Seasonal risk of low pathogenic avian influenza virus introductions into free-range layer farms in the Netherlands. Transbound Emerg Dis. 2020. doi:10.1111/tbed.13649 PMC8048991 32506770

[50] Rohani P, Breban R, Stallknecht DE, et al. Environmental transmission of low pathogenicity avian influenza viruses and its implications for pathogen invasion. Proc Natl Acad Sci USA. 2009;106:10365–10369. doi:10.1073/pnas.0809026106 19497868 PMC2690603

[51] Breban R, Drake JM, Stallknecht DE, et al. The role of environmental transmission in recurrent avian influenza epidemics. PLoS Comput Biol. 2009;5:e1000346. doi:10.1371/journal.pcbi.1000346 19360126 PMC2660440

