# Supplementary figures and images for: Comparative pathogenicity and environmental transmission of recent highly pathogenic avian influenza H5 viruses
Source: Emerg Microbes Infect. 2021 Jan 17;10(1):97–108. doi: 10.1080/22221751.2020.1868274 (PMC7832006; doi:10.1080/22221751.2020.1868274)

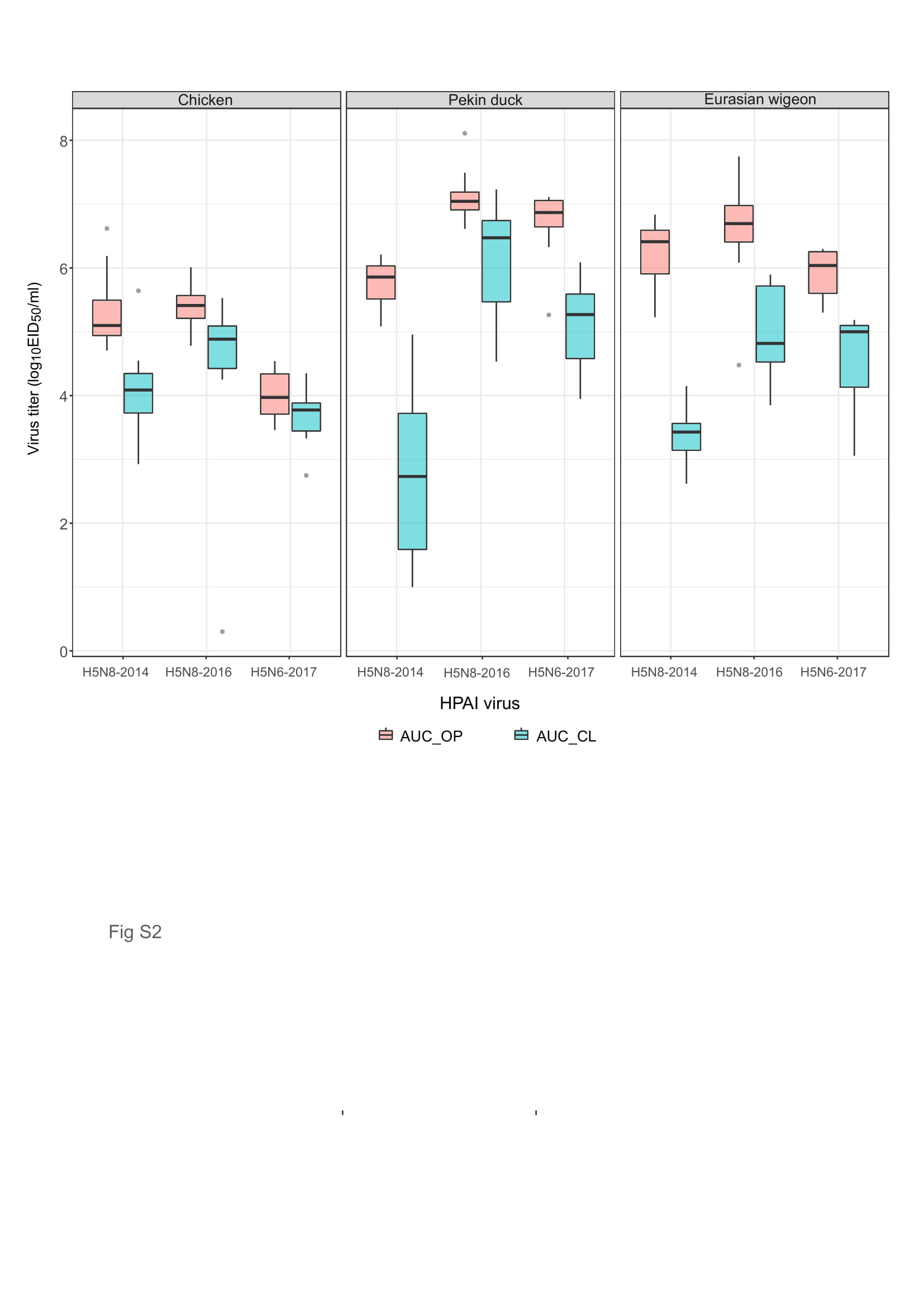

Supplement: FigS2_AUCboxplot_vs2-1_editable.jpg [file TEMI_A_1868274_SM0741.jpg]

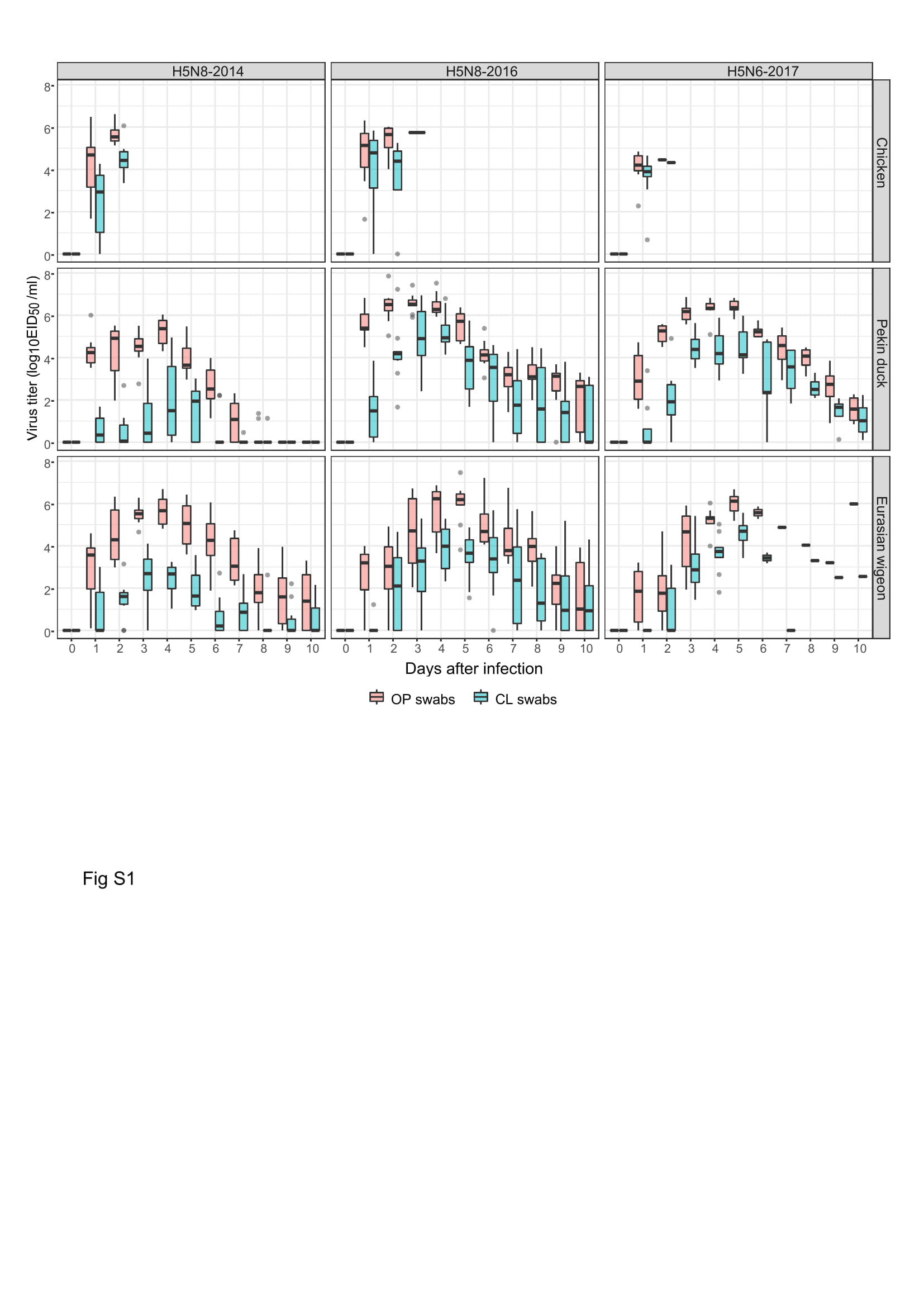

Supplement: FigS1_Shedding_Jose_vs2-1-editable.jpg [file TEMI_A_1868274_SM0740.jpg]
