# Supplementary material for: Comparative pathogenicity and environmental transmission of recent highly pathogenic avian influenza H5 viruses
Source: Emerg Microbes Infect. 2021 Jan 17;10(1):97–108. doi: 10.1080/22221751.2020.1868274 (PMC7832006; doi:10.1080/22221751.2020.1868274)
Supplement: TableS1_mutations_revised.docx [file TEMI_A_1868274_SM0739.docx]

Table S1: Genetic differences between the H5N8-2016 and H5N6-2017 group B viruses.

| segment | # nt | protein | # AA | AA changes | | | | | |
| --- | --- | --- | --- | --- | --- | --- | --- | --- | --- |
| 1 | 204 | PB2 | 6 | E249D | T271V | V292I | A344V | R389K | F678D |
| 2 | 23 | PB1 | 3 | N158S | M195I | T566A |  |  |  |
| 3 | 81 | PA | 6 | R113K | V122I | K158R | L325P | V354I | R615K |
|  |  | PA-X | 4 | R113K | V122I | K158R | A212V |  |  |
| 4 | 19 | HA | 4 | A99D | N113D | M156T | Y503D |  |  |
| 5 | 11 | NP | 1 | F353V |  |  |  |  |  |
| 7 | 13 | MP1 | 3 | F62V | S126C | K134R |  |  |  |
|  |  | MP2 | 4 | K18R | F48L | G66E | D88Y |  |  |
| 8 | 20 | NS | 2 | K39R | M52V |  |  |  |  |

# nt: number of nucleotide differences in the segment

# AA: number of amino acid changes in the protein
